# Human Sensory Neuron-like Cells and Glycated Collagen Matrix as a Model for the Screening of Analgesic Compounds

**DOI:** 10.3390/cells11020247

**Published:** 2022-01-12

**Authors:** Michelle Cristiane Bufalo, Maíra Estanislau Soares de Almeida, José Ricardo Jensen, Carlos DeOcesano-Pereira, Flavio Lichtenstein, Gisele Picolo, Ana Marisa Chudzinski-Tavassi, Sandra Coccuzzo Sampaio, Yara Cury, Vanessa Olzon Zambelli

**Affiliations:** 1 Laboratory of Pain and Signaling, Butantan Institute, São Paulo 05503-900, Brazil; michelle.bufalo@butantan.gov.br (M.C.B.); gisele.picolo@butantan.gov.br (G.P.); 2 Center of Excellence in New Target Discovery, Butantan Institute, São Paulo 05503-900, Brazil; mesalmeida@gmail.com (M.E.S.d.A.); carlos.ocesano@butantan.gov.br (C.D.-P.); flavio.lichtenstein@butantan.gov.br (F.L.); ana.chudzinski@butantan.gov.br (A.M.C.-T.); 3 Laboratory of Pathophysiology, Butantan Institute, São Paulo 05503-900, Brazil; sandra.coccuzzo@butantan.gov.br; 4 Immunogenetics Laboratory, Butantan Institute, São Paulo 05503-900, Brazil; jose.jensen@butantan.gov.br; 5 Innovation and Development Laboratory, Innovation and Development Center, Butantan Institute, São Paulo 05503-900, Brazil; 6 Department of Pharmacology, Institute of Biomedical Sciences, University of São Paulo, São Paulo 05508-220, Brazil

**Keywords:** two-dimensional culture, nociception, inflammatory disease, in vitro assays, analgesic compounds, high-content screening

## Abstract

Increased collagen-derived advanced glycation end-products (AGEs) are consistently related to painful diseases, including osteoarthritis, diabetic neuropathy, and neurodegenerative disorders. We have recently developed a model combining a two-dimensional glycated extracellular matrix (ECM-GC) and primary dorsal root ganglion (DRG) that mimicked a pro-nociceptive microenvironment. However, culturing primary cells is still a challenge for large-scale screening studies. Here, we characterized a new model using ECM-GC as a stimulus for human sensory-like neurons differentiated from SH-SY5Y cell lines to screen for analgesic compounds. First, we confirmed that the differentiation process induces the expression of neuron markers (MAP2, *RBFOX3* (NeuN), and *TUBB3* (β-III tubulin), as well as sensory neuron markers critical for pain sensation (TRPV1, *SCN9A* (Nav1.7), *SCN10A* (Nav1.8), and *SCN11A* (Nav1.9). Next, we showed that ECM-GC increased c-Fos expression in human sensory-like neurons, which is suggestive of neuronal activation. In addition, ECM-GC upregulated the expression of critical genes involved in pain, including *SCN9A* and *TACR1*. Of interest, ECM-GC induced substance P release, a neuropeptide widely involved in neuroinflammation and pain. Finally, morphine, the prototype opiate, decreased ECM-GC-induced substance P release. Together, our results suggest that we established a functional model that can be useful as a platform for screening candidates for the management of painful conditions.

## 1. Introduction

Collagen glycation is frequently observed in neuroinflammatory and neurodegenerative disorders, such as Parkinson’s and Alzheimer’s diseases, osteoarthritis, diabetic neuropathy, amyloid polyneuropathy, and the aging process [1,2]. Glycation is an irreversible and spontaneous process in which proteins, such as collagen, bind to sugar molecules, forming advanced glycation end products (AGEs) [3]. AGEs can activate specific cell surface receptors that transduce signals through downstream proteins, leading to the inflammatory process.

The expression of AGE receptors is cell-type dependent, and the most studied receptors are the receptors for advanced glycation end-products (RAGE), expressed in neuronal cells [4,5]. However, other AGE receptors, including AGE-R1/OST-48/p60, AGE-R2/80K-H/p90, and AGE/Galectin-3, are also found in neuronal cells [4] and participate in AGE signaling.

Collagen glycation modifies the extracellular matrix (ECM) structure and properties, resulting in matrix stiffening, which is frequently observed in osteoarthritic knee joints, for example. This process may affect the optimal biochemical functions and cell–matrix component interactions, potentially disrupting cellular homeostasis [6]. In neurons, ECM-GC-induced tissue damage and inflammation can result in excitation, neuropeptide release, and peripheral sensitization that ultimately contribute to the nociceptive process [7,8].

The glycation of extracellular matrix components affects the neurite extension of neurons in culture. For instance, dorsal root ganglia (DRG) sensory neurons or neuroblastoma cells showed reduced cell attachment and shorter neurite formation after culturing on glycated substrates [9,10,11]. It is well established that cell attachment to the ECM is an important step for neuronal survival and allows a neuronal extension in cell culture. Therefore, ECM disruption due to glycation may interfere with the ability of neurons to extend their neurites.

Importantly, using primary DRG cells, we observed that similar levels of AGEs, to those detected in the arthritic mice serum, reduce neuron interconnection and thin the F-actin filaments, as well as activate pro-nociceptive signaling [12]. However, the use of primary DRG neuronal culture has some drawbacks. In addition to requiring a significant number of animals to obtain enough DRG cells, the purification of a neuron monoculture is difficult due to the presence of non-neuronal cells, such as fibroblasts and satellite cells. Therefore, it is necessary to develop human cell models to enable the screening of large libraries of compounds [13,14].

Here, we characterized the impact of AGEs on nociceptive-like response using a human-derived neuron cell line. We showed that SH-SY5Y cells were successfully differentiated into sensory-like neurons, expressing critical proteins involved in the nociceptive transmission, such as TRPV1, Nav1.7, Nav1.8, and Nav1.9.

Importantly, the incubation of sensory-like neurons on ECM-GC is sufficient to up-regulate c-Fos, a marker for neuronal activity following noxious stimulation and pro-nociceptive gene expressions, as well as induce substance P release. Of interest, the incubation with morphine decreases ECM-GC-induced substance P release, showing that these neurons are responsive to analgesics. Taken together, our results demonstrated that sensory neuron-like cells combined with a glycated collagen matrix are a useful tool for screening analgesic and anti-inflammatory drugs.

## 2. Materials and Methods

### 2.1. Preparation of Glycated Type I Collagen

Collagen type I from rat tail fibrillar state (4.4 mg/mL Corning, New York, NY, USA) was incubated with 200 mM D-ribose, 160 mM D-glucose and 200 mM D-threose (Sigma Aldrich, St. Louis, MO, USA), at 4 °C for 7 days. Glycated collagen (GC) and normal collagen (NC) were digested in 1 mg/mL pepsin and 0.5 M acetic acid (Sigma Aldrich, St. Louis, MO, USA) for 18 h at 37 °C and continuously shaken for collagen solubilization. The pH of the solution was then adjusted to 7.0 and the samples were centrifuged for 5 min at 1500 rpm. Fluorescence was measured in NC and GC supernatants at excitation/emission wavelengths of 360/440 nm for the total AGE quantification and of 328/378 nm for pentosidine determination, using an LS50B luminescence spectrometer (Perkin-Elmer, Waltham, MA, USA) [12,15].

### 2.2. Cell Culture and Neuronal Differentiation

Human neuroblastoma SH-SY5Y cells (ECACC, Sigma Aldrich, St. Louis, MO, USA) were cultured in a 1:1 mixture of Ham’s F12 and Dulbecco’s modified Eagle’s media (DMEM) (Gibco Life Technologies, Grand Island, NY, USA) supplemented with 10% heat-inactivated fetal bovine serum (FBS) (Hyclone Labs, Logan, UT, USA) and 100 U/mL penicillin/streptomycin (Gibco Life Technologies, Grand Island, NY, USA) in a humidified atmosphere of 5% CO_2_ at 37 °C. The medium was changed twice a week, and cells were cultivated at about 80% confluence.

**Figure 1:**
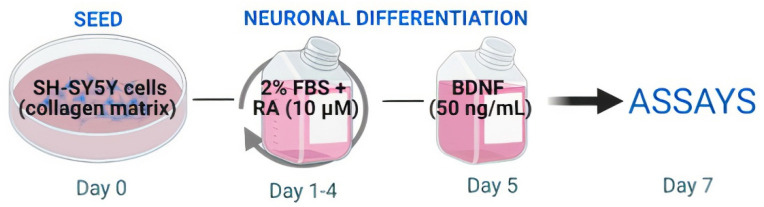
Schematic protocol for neuronal differentiation. On the first day (day 0) cells were plated into normal collagen coated plates. On days 1, 3, and 4, the medium was removed and the cells were incubated with a differentiation medium containing retinoic acid (RA—10 µM). On day 5 the medium was removed and the culture was stimulated with a medium containing brain derived neurotrophic factor (BDNF—50 ng/mL). On day 7, differentiated neurons were used in the experiments.

Cells were not cultured beyond passage 20. In our neuronal differentiation protocol (Figure 1), 5 × 10^4^ cells/well were seeded in normal collagen matrix coated plates (NC-100 µg/mL). After 24 h (day 1), the medium was replaced with FBS reduced to 2% (differentiation medium), and supplemented with 10 µM all-trans retinoic acid (RA, Sigma Aldrich, Saint Louis, MO, USA). Cells were incubated for 5 days, with daily medium replacement, except for the second day. On day 5 of differentiation, cultures were stimulated with serum-free medium supplemented with human brain-derived neurotrophic factor (BDNF 50 ng/mL—R&D Systems, MN, USA). On day 7 of differentiation, neurons were used in the experiments [16,17].

### 2.3. Validation of the Sensory Neuron-like Cells Differentiation Process

Human neuroblastoma SH-SY5Y cells were plated on normal collagen (NC)-coated coverslips and subjected to neuronal differentiation as described previously. Cells were fixed for 30 min at room temperature in 3.7% paraformaldehyde (Sigma-Aldrich, Saint Louis, MO, USA) in phosphate-buffered saline (PBS, Sigma-Aldrich, Saint Louis, MO, USA) at pH 7.4. After washing in PBS, cells were permeabilized for 5 min in 0.5% Triton X-100 in PBS (Sigma-Aldrich, Saint Louis, MO, USA) and washed three times for 10 min, also in PBS.

Samples were blocked for 1 h at room temperature with 3% bovine serum albumin—BSA (Sigma-Aldrich, Saint Louis, MO, USA) in PBS. Primary antibodies were diluted in PBS/1% BSA (chicken anti-neuron specific β-III tubulin 1:500; Merck Millipore, Burlington, MA, USA; mouse anti-MAP2 1:800; Abcam, Waltham, MA, USA; mouse anti-NeuN 1:800; Abcam, MA, USA; rabbit anti-TRPV1 1:200 Santa Cruz Biotechnology, MA, USA; rabbit anti-Nav1.7 1:200; rabbit anti-Nav1.8 1:200; and rabbit anti-Nav1.9 1:200, Abcam, Waltham, MA, USA) and incubation was carried out overnight at 4 °C.

Then, cells were incubated with secondary PE-conjugated goat anti-chicken antibodies (1:500), and Alexa Fluor 647-conjugated goat anti-rabbit (1:300) for 1 h at room temperature (Cell Signaling Technology, Danvers, MA, USA). Negative controls were incubated with secondary antibodies. Nuclei were stained with Hoechst-33342 (1:3000) (Gibco Life Technologies, Grand Island, NY, USA). Cells were examined using a laser scanning confocal microscope (Zeiss—Leica, Oberkochen, Germany) and High Content Screening (HCS—Molecular Devices, San Jose, CA, EUA). The same immunostaining was performed with the SH-SY5Y cell line. The percentage of increase of each neuronal marker was calculated considering the ratio between the immunostaining in the sensory neuron-like cells and the undifferentiated SH-SY5Y cells.

### 2.4. Cell Viability

Sensory neuron-like cells were incubated with either culture medium alone (control), 100 µg/mL of ECM-NC or ECM-GC, or ECM-NC (100 µg/mL) plus interleukin-1β (IL-1β, 10 ng/mL as a positive control) for 72 h. Supernatants were removed to evaluate the effects of the treatments on cellular viability using fluorescence-based Live/Dead Cell Viability Assays (Thermo Fisher Scientific, Waltham, MA, USA) and MTT assay. Cells were incubated with the combined Live/Dead assay reagents for 30 min at room temperature and then, the reagents were removed and washed three times in PBS. Fluorescence was evaluated using HCS.

A time-course evaluation was performed to confirm the non-cytotoxicity of the treatments, then, cell cultures were incubated with ECM-NC, ECM-GC, or ECM-NC plus interleukin-1β during 1 min, 45 min, 2 h, 6 h, 18 h, 24 h, 48 h, and 72 h. Supernatants were removed after each time of incubation and cells were incubated with 3-(4.5-Dimethylthiazol-2-yl)-2,5-diphenyl tetrazolium bromide (MTT, 0.5 mg/mL in culture medium, Sigma-Aldrich, St. Louis, MO, USA) for 3 h [18]. Next, the solution was removed, and dark blue formazan crystals were solubilized with 100 µL dimethylsulfoxide (DMSO, Merck, Darmstadt, Germany) for 10 min. Quantification of formazan was performed using an ELISA automatic microplate reader (SLT, Wolfurt, Austria) at 570 nm and data are expressed as % of control.

### 2.5. Immunofluorescence Detection of Receptors for AGEs Using HCS Equipment

Sensory neuron-like cells were incubated with ECM-NC, ECM-GC, or ECM-NC matrix with IL-1β for 24 h to detect AGEs receptor expression. Then the cells were fixed, permeabilized, and blocked as described previously. Cells were incubated with primary antibodies diluted in PBS and 3% BSA: chicken anti-neuron specific β-III tubulin diluted 1:500 (Merck Millipore, Burlington, MA, USA) and rabbit anti-RAGE diluted 1:200 (Abcam, Waltham, MA, USA); or incubated with chicken anti-neuron specific β-III tubulin diluted 1:500 and rabbit anti-Galectin-3 (1:500, Abcam, Waltham, MA, USA) overnight at 4 °C.

Cells were then incubated with secondary PE-conjugated goat anti-chicken antibodies (1:500 dilution), and Alexa Fluor 647-conjugated goat anti-rabbit (1:300), for 1 h at room temperature. Negative controls were incubated with secondary antibodies. Nuclei were stained with Hoechst-33342 (1:3000), and the fluorescence was determined using a Confocal Microscope (Zeiss) and images were captured with a confocal laser-scanning microscope (Confocal TCS, SP8, Leica, Wetzlar, Germany).

### 2.6. c-Fos Transcription Factor Expression Using HCS Analysis

Sensory neuron-like cells were incubated with culture medium only (control), ECM-NC, ECM-GC, and ECM-NC plus IL-1β for 1 h, and then fixed and permeabilized as described previously. The cFos transcription factor protein was detected with rabbit anti-Fos primary antibody diluted 1:250 in PBS and 3% BSA (Sigma-Aldrich, Saint Louis, MO, USA) overnight at 4 °C under agitation. After this period, cells were washed three times with PBS and then incubated with secondary Alexa Fluor 647-conjugated goat anti-rabbit diluted 1:300 for 1 h at room temperature, protected from light (Cell Signaling Technology, Danvers, MA, USA). Fluorescence was determined using an HCS (Molecular Devices).

The HCS was used to acquire 16 images per well at 20× magnification. Cell quantification based on images was performed according to the following steps. The nuclei were automatically identified within illumination-corrected Hoechst-33342 to define nuclear boundaries: the intensity above the local background was used for finding the nuclei. An internal mask (cytoplasm) was defined by dilating the nuclear mask out to the edge of the Hoechst-33342; the intensity above the local background for the stained area was used to distinguish positive from negative cells. We measured the fluorescence intensity parameters of the transcription factor inside the nuclear and cytoplasm area. The quantitative data shown represent the fluorescence intensity of protein relative to the control (secondary antibody).

### 2.7. SCN9A (Nav1.7) and TACR1 (NK1) Gene Expression—Real-Time Quantitative PCR

To perform a time-series analysis for pain and inflammatory gene expression, 1 × 10^6^ SH-SY5Y cell-derived neurons were plated, subjected to neuronal differentiation, and incubated with ECM-NC, ECM-GC, ECM-NC plus IL-1β, or culture medium alone (control), for 1 min, 45 min, 2 h, 6 h, 18 h, 24 h, 48 h and 72 h. After each period of incubation, RNA was extracted using the RNeasy Mini Kit (Qiagen, Hilden, Germany). The final RNA concentration and purity were determined using a NanoDrop spectrophotometer (ThermoFisher Scientific, Madrid, Spain).

RNA quality was determined with the Agilent Bioanalyzer 2100 using the RNA Nano 6000 kit (Agilent, Waldbronn, Germany), and samples were only used with a minimum RNA integrity number (RIN) of 8. One µg of RNA was reverse transcribed with the GoScript Reverse Transcriptase (Promega, Madison, USA) and oligo dT18 primers. Amplification mixtures contained 2 µL template cDNA, 5 µL Fast SYBR Green PCR Master Mix (Invitrogen, Waltham, MA, USA), and 0.2 mM specific primers, resulting in a total volume of 10 µL.

PCR cycling was carried out using the StepOnePlus Real-Time PCR detector (Applied Biosystems, Waltham, MA, USA), using default cycling conditions: 95 °C for 20 s (1 cycle) followed by 95 °C for 3 s and 60 °C for 30 s (40 cycles), and melt curve (95 °C for 15 s, 60 °C for 1 min and increments of 0.3 °C up to 92 °C). The human hypoxanthine-guanine phosphoribosyltransferase (*HPRT1*) and cyclophilin A (*PPIA*) genes were selected with the GeNorm VBA applet [19] for use as reference genes. The relative level of mRNA was calculated using the comparative cycle threshold (CT) method and expressed as 2^−ΔΔCT^.

Target genes belonging to pain transduction and pain transmission were chosen and included: *SCN9A* (Nav1.7) sodium channel that plays a role in nociception signaling) and *TACR1* (NK1, neurokinin receptor for substance P). Primer-pair sequences are described in Table 1 and were designed with Primer-Blast (Source: https://www.ncbi.nlm.nih.gov/tools/primer-blast/ (accessed on 5 August 2019).

### 2.8. Neurite Growth Analysis by the HCS Assay

Neuron-like cell cultures were incubated with ECM-NC, ECM-GC, ECM-NC plus IL-1β, or culture medium (control). Briefly, after 72 h, the supernatants were aspirated, cells were washed with PBS 1× and then fixed for 1 h with cold 3.7% PFA and washed again with PBS 1×. Cells were blocked with 1% BSA for 30 min and then incubated overnight at 4 °C with primary neuron-specific anti-β-III tubulin (1:500; AB9354, Merck Millipore). After three washes, cells were incubated with Hoechst-33342 (1:3000; Thermo Fisher Scientific) and goat anti-chicken IgG (H + L) secondary antibody PE conjugate (1:1000; AP503H, Merck Millipore) for 1 h at room temperature.

Finally, the plates were subjected to high-content imaging analysis (HCS) using the MetaXpress High-Content Image Acquisition & Analysis Software (Molecular Devices). Automated HCS assay was used to quantify and correlate neuron and neurite morphology. The quantification of neurite parameters was obtained by HCS and MetaXpress software according to the following steps:

(1) The cell mask to identify the size of cell bodies was defined by the approximate max width of the cells. The fluorescence intensity above the local background was used to define positive or negative cells related to the β-III tubulin marker. An additional criterion was used to separate cell bodies from branch points (thick outgrows).

(2) The total amount associated with the cell body was defined by the max-width used to distinguish the beginning of outgrowth from cell bodies or branch points. The fluorescence intensity above the local background was used to measure the intensity for outgrowth just as for the cell bodies. The minimal cell growth to log as significant was used for scoring cells as positive or negative for outgrowth.

(3) The number of cells was obtained by the nuclear mask defined with Hoechst-33342.

(4) The total outgrowth is the total length of skeletonized outgrowth in µm (correlated for diagonal lengths). The system was used to acquire 16 images per well at 20× magnification and the calculated data represent the mean value of the neurite outgrowth in each condition (control, ECM-NC, ECM-GC and ECM-NC plus IL-1β).

### 2.9. Analysis Substance P and β-Endorphin Release—Muliplex Assay

Sensory neuron-like cells were incubated with ECM-NC, ECM-GC, ECM-NC plus IL-1β or culture medium (control), for 1 min, 45 min, 6 h, 12 h, 24 h, 48 h, and 72 h. After incubation, supernatants were collected, and substance P and β-endorphin release were measured with the Milliplex Map Kit Human Neuropeptide Magnetic Bead Panel (Cat. HNPMG-35K, Merck Millipore) according to the manufacturer’s instructions. Median fluorescence intensity (MFI) was evaluated with a MAGPIX Luminex reader (Merck Millipore).

To validate the model, morphine was used as a control. Sensory neuron-like cells were incubated with culture medium (control), ECM-GC, or morphine (10 µM, dissolved in medium culture), for 1 h. Then, the ECM-GC was removed from the wells and replaced with medium culture or morphine. After 24 h of incubation, the supernatants were collected, and substance P was evaluated.

### 2.10. Statistical Analysis

Graphs and statistical analysis were performed using GraphPad Prism^®^ 8 and also using Python (Numpy and Scipy). Data were analyzed using *t*-test or one-way ANOVA or two-way ANOVA with Tukey or Dunnett post hoc tests or, depending on the experiment. The results are expressed as the mean ± SEM (standard deviation of the mean). A *p*-value of *p* < 0.05 was considered significant.

## 3. Results

### 3.1. Confirmation of Glycation Collagen Process

**Figure 2:**
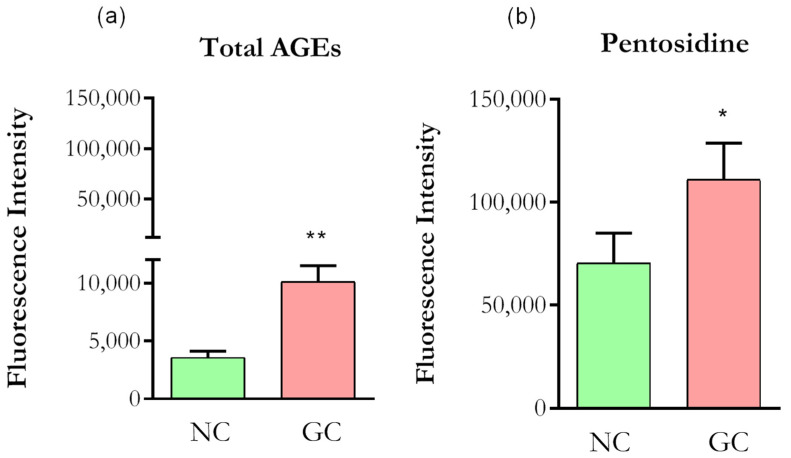
Autofluorescence of collagen. (**a**) Optical density (DO) values were taken from 370/440 nm for Total AGEs. (**b**) DO values were taken from 328/378 nm for Pentosidine. NC (normal collagen), GC (glycated collagen). Data were analyzed by *t*-test, * *p* < 0.01; ** *p* < 0.001). Five independent and similar assays were performed in triplicate.

As AGEs exhibit strong autofluorescence, we first confirmed the formation using confocal microscopy. Collagen incubation with 200 mM D-ribose, 160 mM D-glucose and 200 mM D-threose for seven days resulted in a significant increase in fluorescence intensities of both total AGEs and pentosidine compared to normal, non-glycated collagen (Figure 2a,b), confirming that the glycation protocol produced highly modified collagen.

### 3.2. SH-SY5Y Differentiation into Sensory-like Neuron Culture 

**Figure 3:**
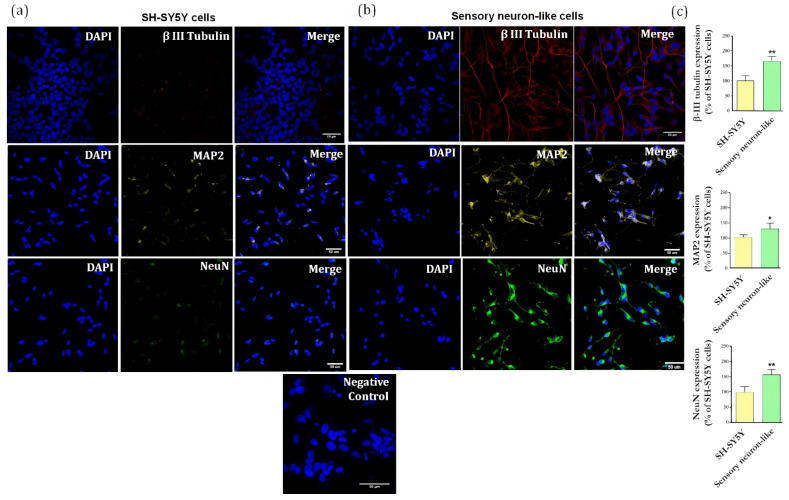
(**a**) Immunofluorescence of specific neuronal markers (β-III tubulin, MAP2 and NeuN) in undifferentiated cells (SH-SY5Y cells) and (**b**) differentiated cells (sensory neuron-like cells). Representative images of five areas. Negative control of immunostaining. Scale bar: 50 µm. (**c**) Relative quantification of immunostaining for βIII-tubulin, MAP2, NeuN representing the ratio expression in the sensory neuron-like cells and SH-SY5Y cells. The data were analyzed by *t*-test, * *p* < 0.05; ** *p* < 0.001). The data represent three independent and similar assays.

Confocal images demonstrate that the protocol of neuronal differentiation produces perceptible changes in cell morphology as compared to SH-SY5Y cells (Figure 3a,b). The differentiated cells (sensory neuron-like cells) show the formation of a rounded cell body that projects an extensive network of neurofilaments. They form branches of more elongated neurites projections connecting surrounding neurons (Figure 3b).

**Figure 4:**
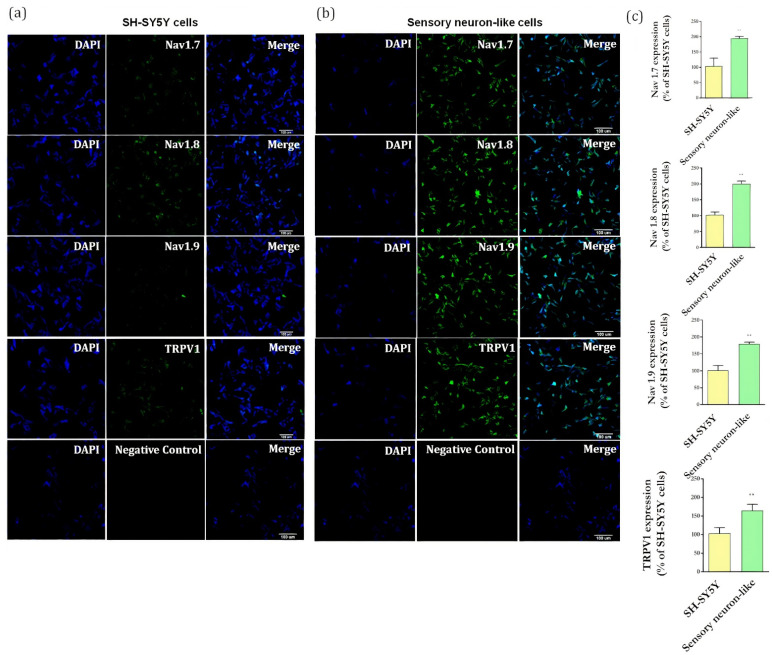
(**a**) Representative images of immunofluorescence of Nav1.7, Nav1.8, Nav1.9, and TRPV1 markers in undifferentiated cells (SH-SY5Y cells) and (**b**) in differentiated cells (sensory neuron-like cells). Magnification: 20×. Negative controls of immunostaining. Scale bar: 100 µm. (**c**) Quantification of Nav1.7, Nav1.8, Nav1.9, TRPV1 expression representing the ratio between the immunostaining in the differentiated cells (sensory neuron-like cells) and the in undifferentiated cells (SH-SY5Y cells). The data were analyzed by *t*-test, ** *p* < 0.001). The data represent three independent and similar assays.

Importantly, the staining for βIII-tubulin (a major constituent of microtubules formation within the cytoskeleton), microtubule associated protein 2 (MAP2, neuron-specific cytoskeletal protein enriched in dendrites) and neuronal nuclear protein (NeuN) clearly shows that all cells display a different pattern and intensity of protein expression when compared to the undifferentiated cells. Figure 4c shows the ratio of increase for each protein compared to the undifferentiated cells: βIII-tubulin (67%), MAP2 (40%), NeuN (58%), and TRPV1 (64%).

It is well established that RA-induced differentiation promotes upregulation in neuronal markers [20] and importantly, these neurons showed an increase in expression of channels involved in pain processes, including TRPV1, Nav1.7, Nav1.8, Nav1.9 (Figure 4b), when compared with undifferentiated cells (Figure 4a). These results showed that the differentiation protocol resulted in neurons expressing markers typically found in peripheral sensory neurons.

### 3.3. Cell Viability and AGEs Receptors (AGER (RAGE) and LGALS3 (Galectin-3) Expression

**Figure 5:**
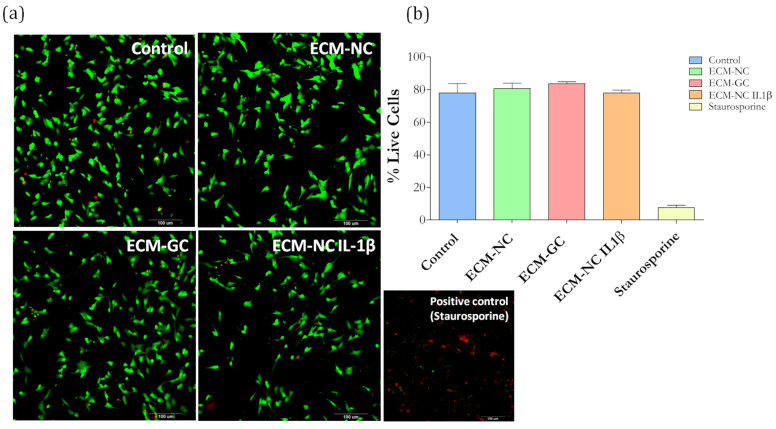
(**a**) Cell viability images show dead cells stained in red and live cells stained in green. Scale bar: 100 µm. (**b**) Cellular viability quantification (% of live cells) performed at 72 h. ECM-NC, ECM-GC, ECM-NC IL-1β, and positive control (staurosporine, 5 µM). Three independent and similar assays were performed in triplicate. Data were analyzed by one-way ANOVA with post hoc testing by Dunnett *p* > 0.05).

We first checked whether the ECM-GC interferes with the cell viability. Sensory neuron-like cells incubated with ECM-NC, ECM-GC, or ECM-NC IL-1β for 72 h display a similar percentage of living cells as compared to control cells, incubated with medium culture alone (Figure 5a,b). The cell viability was also confirmed by the MTT method that was performed in several time points (1 min, 45 min, 2 h, 6 h, 18 h, 24 h, 48 h, and 72 h) (Supplementary Material Figure S1).

**Figure 6:**
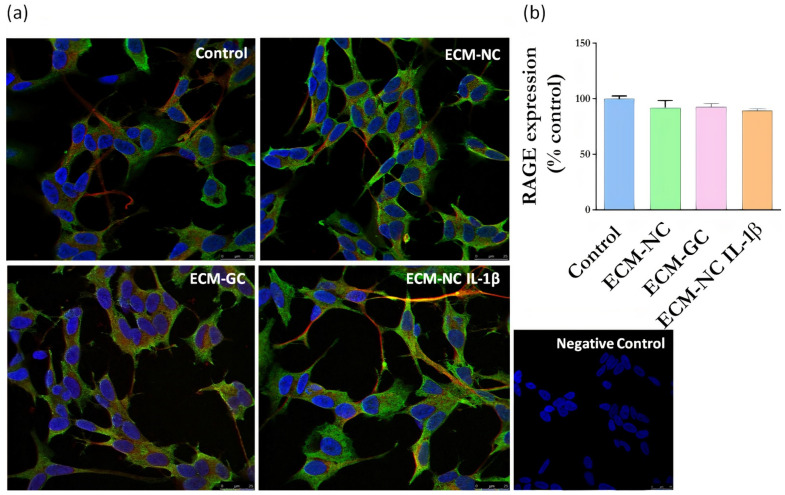
(**a**) Representative images of immunofluorescence of RAGE expression. Neuronal class β-III tubulin (red) 1:500; Hoechst-33342 was used to stain nuclei (blue) 1:3000; RAGE receptor (green) 1:200 in control cells (differentiated/ without ECM) and cells treated with ECM-NC, ECM-GC, ECM-NC IL-1β. Negative control of immunostaining. Magnification 40×. Scale bar: 25 µm. (**b**) Relative quantification of immunostaining for RAGE representing the ratio between the experimental and the control group. The data were analyzed by *t*-test. The data represent three independent and similar assays. Data were analyzed by one-way ANOVA with post hoc testing by Dunnett *p* > 0.05).

**Figure 7:**
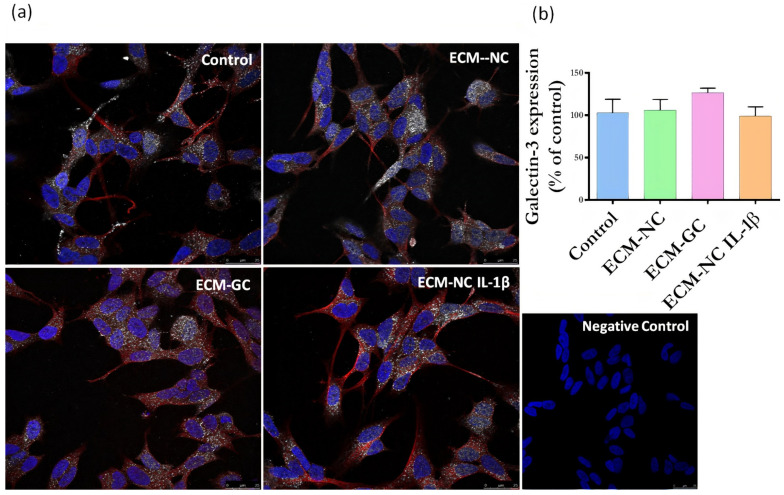
(**a**) Representative images of immunofluorescence of Galectin-3 expression. Neuronal class β-III tubulin (red) 1:500; Hoechst-33342 was used to stain nuclei (blue) 1:3000; Galectin-3 receptor (green) 1:200 in control cells (differentiated/ without ECM) and cells treated with ECM-NC, ECM-GC, ECM-NC IL-1β. Negative control of immunostaining. Magnification 40×. Scale bar: 25 µm. (**b**) Relative quantification of immunostaining for Galectin-3 representing the ratio between the experimental and the control group. The data were analyzed by *t*-test. The data represent three independent and similar assays. Data were analyzed by one-way ANOVA with post hoc testing by Dunnett *p* > 0.05).

Next, we evaluated whether sensory neuron-like cells express AGE receptors. The neurons express RAGE (Figure 6) and Galectin-3 receptors (Figure 7), regardless of incubation with ECM-GC or ECM-NC IL-1β. The percentage of cells expressing RAGE and Galectin-3 receptors is 80–100%.

### 3.4. GC Matrix Activates Sensory Neurons-like Cells

**Figure 8:**
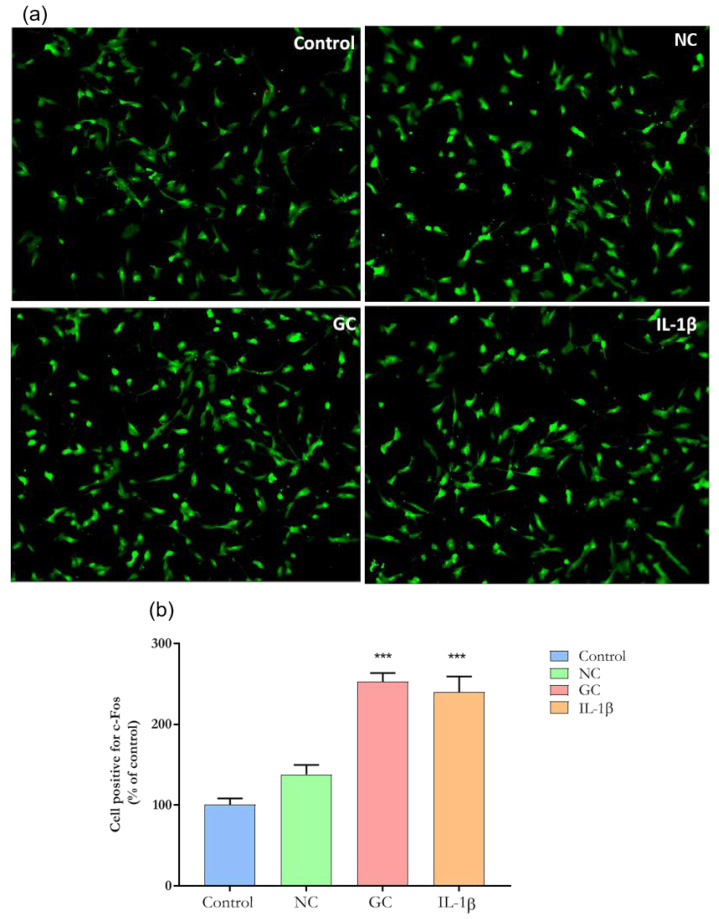
Representative images of immunofluorescence of c-Fos expression. (**a**) c-Fos expression in sensory neuron-like cells incubated with ECM-NC, ECM-GC, ECM-NC IL-1β or medium culture for 1 h. Magnification: 20×. (**b**) Quantification of c-Fos nuclear expression (% of control). Data were analyzed by one-way ANOVA with post hoc testing by Dunnett (*** *p* < 0.0001 different from Control). NC (normal collagen) and GC (glycated collagen). Three independent and similar assays performed in triplicate.

AGE formation has been associated with increased inflammation [4]. However, whether the glycation process directly activates neurons is unknown. *C-Fos* is a proto-oncogene that is expressed within some neurons following depolarization. The protein product, c-Fos protein, can be identified by immunohistochemical techniques [21]. As shown in Figure 8a,b, cells incubated with either ECM-GC matrix or IL-1β display increased c-Fos expression when compared to cells incubated with NC or cell medium alone (control). This data suggest that the glycation was sufficient to activate sensory-like neurons.

### 3.5. Effect of GC on SCN9A (Nav1.7) and TACR1 (NK1) Genes

**Figure 9:**
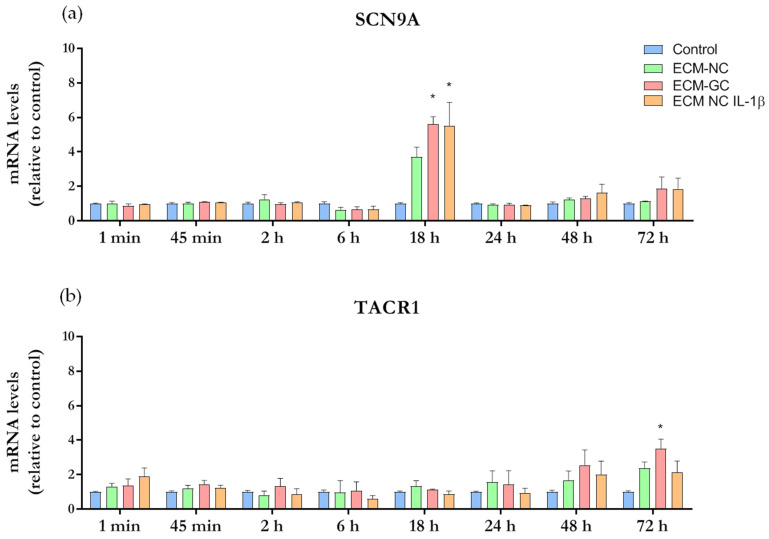
Time course of gene expression analysis. Sensory neuron-like cells were incubated with media culture (control), ECM-NC or ECM-GC or ECM-NC plus IL-1β for 1 min, 45 min, 2 h, 6 h, 18 h, 24 h, 48 h, and 72 h. Gene expressions were normalized by *PPIA* and *HPRT1* housekeeping genes. Data were analyzed by two-way ANOVA with post hoc testing by Tukey (* *p* < 0.05 different from control). NC (normal collagen), GC (glycated collagen). Three independent and similar assays were performed in triplicate. (**a**) SCN9A and (**b**) TACR1.

Nav1.7 and NK1 receptors ligand are involved in the induction and maintenance of pain state. The results presented in Figure 9a show that ECM-GC and IL-1β significantly increased SCN9A gene expression at 18 h incubation, when compared to control. Of interest, the incubation of neuron-like cells with ECM-GC, but not with ECM-NC plus IL-1β, upregulated TACR1 expression at later stages (72 h) of the study (Figure 9b), indicating that these neurons become hypertensive along the time.

These results can be seen as a log fold change (LFC) heatmap in Supplementary Material Figure S2. Cell colors represent the mean of LFC for ECM-GC, ECM-NC, and ECM-NC plus IL-1β. Here, we highlight the following upregulation results for ECM-GC: SCN9A 5.62 at 18 h [5.14, 6.10], and TACR1 showed a later ECM-GC upregulation at 72 h, 3.52 [2.91, 4.13].

All results represent the mean LFC followed by the confidence interval (in brackets), the last was calculated using a confidence level of 70%. LFC (ECM-GC/Control) minus LFC (IL-1β/Control) heatmap (Supplementary Material Figure S2) presented low values of LFCs, and we inferred that ECM-GC is a good pro-inflammatory stimulus to induce SCN9A and TACR1 gene expression (Supplementary Material Figure S3).

### 3.6. Neurite Growth Inhibition by GC Matrix

Physiological neuronal function depends on the formation of a neurite network [22], which is guided by the cytoskeleton [23]. Modifications in network organization lead to altered pain-associated signal transduction and electrical activity of sensory nerves [24]. Then, in order to further characterize the effect of the glycated matrix on neurons, we evaluated the neurite outgrowth.

**Figure 10:**
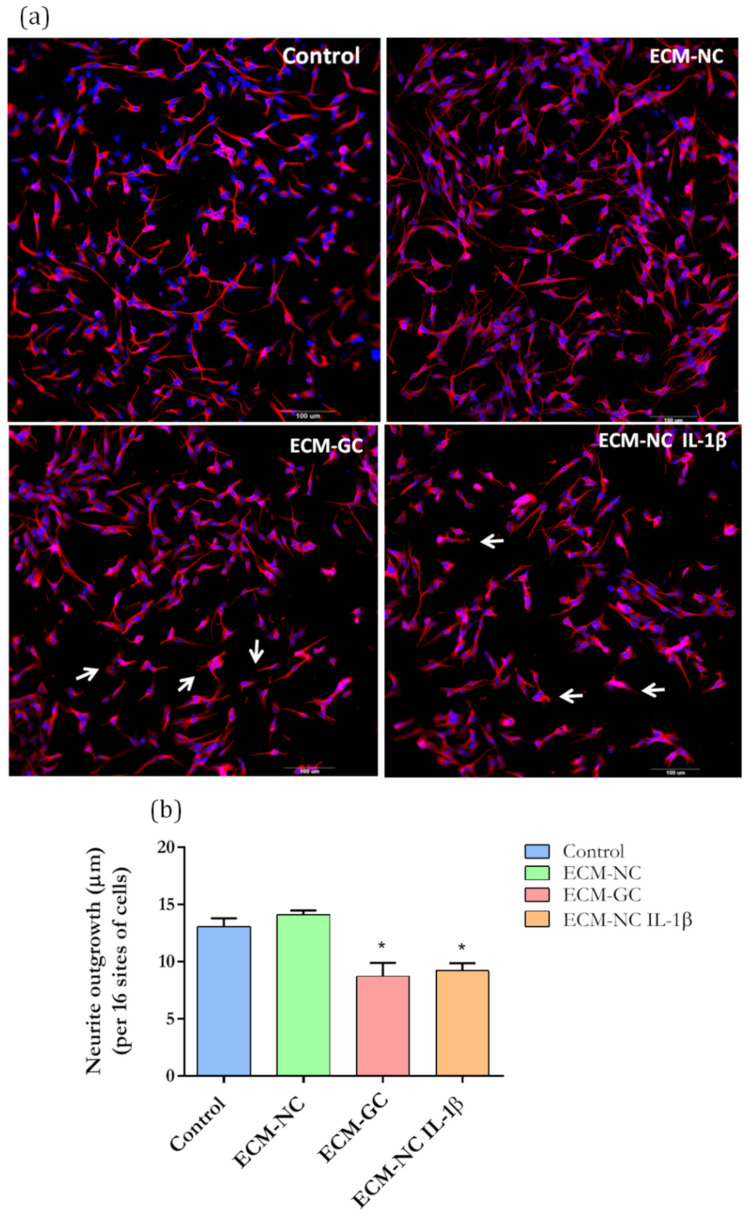
(**a**) Representative images of immunofluorescence show neurite length in sensory neuron-like cells. Arrows represent the reduced neuron interconnection and retraction of neurons outgrowth. (**b**) Quantification of neurite length in neuron-like cells based on β-III tubulin immunostaining. Data from 72 h of incubation. Magnification 20×. Scale bar: 100 µm. Data were analyzed by *t*-test (* *p* < 0.05 different from control), ECM-NC (normal collagen), ECM-GC (glycated collagen). Three independent and similar assays were performed in triplicate.

Incubation with either ECM-GC or ECM-NC IL-1β for 72 h (the last stage in the time series of our study) significantly inhibited neurite growth when compared to control and EMC-NC (Figure 10a,b). Our results suggest that AGEs may interfere with cytoskeleton filaments, resulting in the reduced neuronal interconnection of sensory neuron-like cells, up-regulating pro-nociceptive signaling pathways.

### 3.7. The Collagen Glycation Functionally Activates Sensory-like Neurons

**Figure 11:**
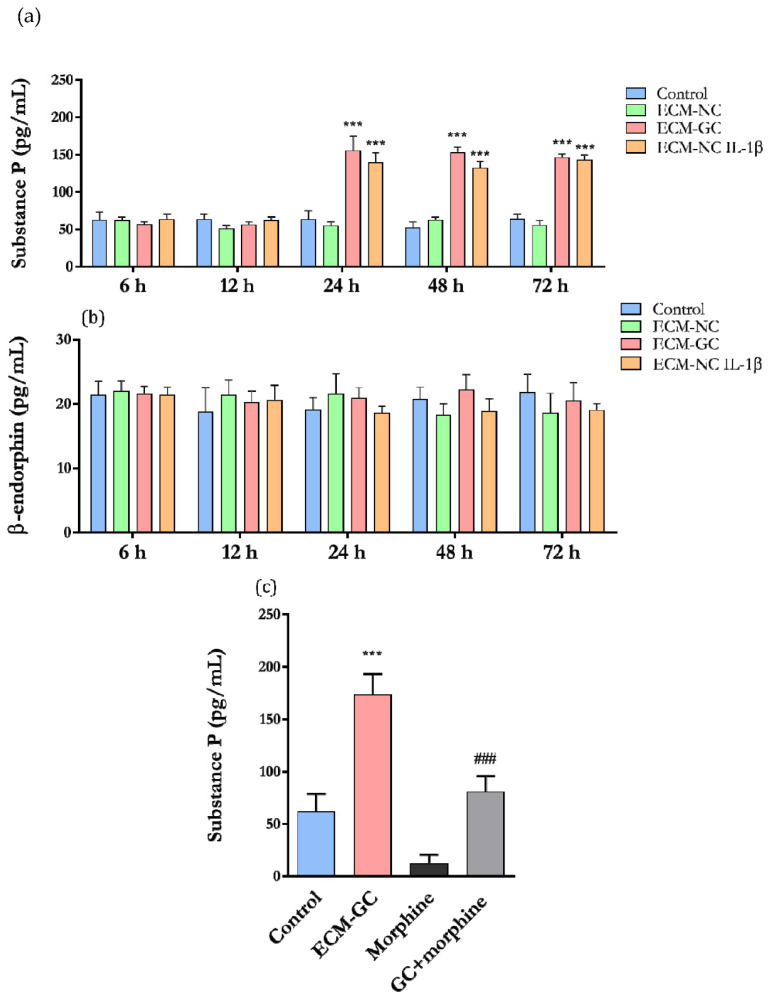
(**a**) Substance P and (**b**) β-endorphin release by neuron-like cells after incubation with ECM-NC or ECM-GC or ECM-NC plus IL-1β for 6, 12, 24, 48, and 72 h; data were analyzed by two-way ANOVA with post hoc testing by Dunnett (*** *p* < 0.0001 different from control). (**c**) Cells were incubated with morphine (10 µM) for 24 h, and the substance P levels were evaluated. Data were analyzed by one-way ANOVA and Tukey (*** *p* < 0.0001 different from control and ^###^ *p* < 0.0001 different from GC). NC (normal collagen) and GC (glycated collagen). Three independent and similar assays were performed in triplicate.

Considering that substance P is a neurotransmitter highly expressed in the primary sensory neurons and leads to neurogenic inflammation [25], we functionally characterized the effect of ECM-GC by evaluating the neuropeptide release. As shown in Figure 11a, substance P secretion is significantly increased in neurons incubated with ECM-GC as well as ECM-NC plus IL-1β, from 24 to 72 h, compared to the control.

β-endorphin is an opioid receptor agonist producing analgesic effects [26]. ECM-GC and ECM-NC plus IL-1β did not induce the release of β-endorphin and maintained the same level of a few picograms (Figure 11b). Therefore, the ECM-GC preferentially activates pro-nociceptive pathways on neurons. No difference in substance P and β-endorphin release was observed at 1 and 45 min (data not shown).

In order to confirm that our model efficiently detects compounds with antinociceptive properties, we next checked whether these neurons respond to the morphine action. Since ECM-GC induced the release of substance P, we detected that morphine significantly reduced ECM-GC-induced substance P secretion (Figure 11c). We believe that these results confirm the hypothesis that this model is a useful platform for screening pharmacological candidates for the management of painful conditions.

## 4. Discussion

Increased collagen-derived advanced glycation end-products (AGEs) contribute to the pathogenesis of painful chronic diseases, such as osteoarthritis, diabetic neuropathy, and neurodegenerative disorders. Considering that pain affects one in five individuals globally [27], it is urgent to find new analgesics and/or molecular pathways involved in nociception.

Immortalized-derived cell culture provides a physiologically relevant system to mimic some diseases, as well as for the discovery of drugs with therapeutic potential. Here, we showed that ECM-GC interacting with sensory-like cells mimics a painful microenvironment, providing a useful model to study new analgesics molecules. Previously, we developed and characterized an in vitro ECM-GC [12] and showed that ECM-GC was sufficient to activate the nociceptive signaling in primary sensory neurons [12]. In this study, we provide an alternative for the use of rodents and further characterized phenotypic changes in response to AGEs.

First, we confirmed that the differentiation protocol produces a uniform population of mature neuronal cells expressing key proteins also detected in primary neuronal cultures, such as β-III tubulin, MAP2 and NeuN [28,29]. Importantly, undifferentiated human SH-SY5Y cells rapidly proliferate and express low quantities of mature neuron markers. Studies demonstrate that, at the end of the differentiation process, to arrest the cell growth cycle and synchronize cells, the expression of proliferative transcription factors are downregulated, which closely correlates to the loss of tumor features [30]. Further experiments are necessary to investigate whether this process occurs in our differentiated cells.

We also demonstrated that the differentiation process stimulates the expression of receptors/channels essential for the nociceptive signaling pathway, including TRPV-1, Nav1.7, Nav1.8, and Nav1.9 [16,28,30,31]. Accordingly, Yin et al. [13] reported that undifferentiated SH-SY5Y cells lack mRNA for nociceptor markers such as, TRPV1, CGRP, substance P (TACR1), corroborating our data showing that the differentiation process is required for expressing proteins found in primary sensory neurons.

Next, we examined whether the sustained glycation process is sufficient to alter the expression of genes involved with nociception. Of interest, ECM-GC upregulated the SCN9A gene, which encodes Nav1.7, a channel highly expressed in nociceptors that contribute to the generation and maintenance of pain signals [32]. This result suggests that ECM-GC may favor hyperexcitability and the synaptic transmission, a critical phenomenon in pain development [33,34]. Moreover, TACR1 (gene for NK1 receptor) is upregulated at 72 h. This is a neurokinin receptor that mediates the substance P effects, and is linked to a variety of physiological and biological processes including the regulation of neurotransmission, pain and inflammation [35].

Once the neuronal markers were characterized and considering that ECM-GC disrupts cellular function and tissue homeostasis [36], we next showed that ECM-GC does not disturb the cellular viability. Of interest, RAGE and Galectin-3 receptors are expressed in the cell surface regardless of the condition. These receptors function as signal transduction receptors for AGEs and its activation results in nuclear factor kappa B (NF-κB) transcription and inflammatory signaling pathway activation [37].

Of interest, the importance of RAGE goes beyond the glycation process. Studies demonstrate that LPS-induced DRG sensitization requires functional expression of RAGE. Therefore, LPS-mediated regulation of neuroinflammatory responses can be potentiated by the glycation process. Moreover, DNA-binding proteins (HMGB-1), secreted during the hyperalgesic phases of inflammatory disorders, may also interact with RAGE and activate intracellular signaling pathways and inflammatory response [33,38].

GC-derived AGEs upregulate c-Fos mRNA expression and activate the TGF-β signaling pathway in mesangial cells, exerting a variety of effects on cell proliferation, cell differentiation, and embryogenesis [34]. Despite these findings, whether ECM-GC activates c-Fos transcription factor in neurons was unknown. In our study, both ECM-GC and ECM-NC plus IL-1β significantly upregulated c-Fos expression. It is well known that following the transcription of c-Fos, the mitogen-activated protein kinase (MAPK) signaling pathway is activated, and neuropeptides are released [39].

In this regard, our group previously demonstrated that ECM-GC triggers p38 MAPK and ERK activation in two-dimensional primary DRG neuron cultures and induces inflammatory mediator release, such as nitric oxide (NO) and tumor necrosis factor alpha (TNF-α) [12]. Then, considering that AGEs induce neuronal activation by increasing c-Fos expression in human sensory neuron-like cells and stimulate the rapid phosphorylation of ERK in DRG primary culture, following the neuropeptides and inflammatory mediator release that contribute to pain processes, the study of these markers is a good parameter to screening analgesic molecules.

As mentioned before, the neuronal functions depend on the formation of a neurite network [22], which is guided by the cytoskeleton [23]. Changes in the neuronal network interfere with neuron-to-neuron communication and the synaptic reorganization, resulting in long-term changes in network hyperexcitability [24,40,41]. Our study showed that ECM-GC reduces neurite outgrowth, and a similar response was observed after incubation with ECM-NC plus IL-1β.

Consistent with these observations, the reduction of neuron interconnection was reported previously by our group, when rat DRG neurons were cultured in a ECM-GC two-dimensional coating system [12]. This data suggests that AGE modifications reorganize the neuronal cytoskeleton filaments with subsequent neurite retraction allowing the cell adaptation to the pro-nociceptive microenvironment [42]. Of interest, neurite retraction is also observed in age-related neurodegenerative diseases, which may be associated with high levels of AGEs and accumulation of the modified proteins, which in turn sustain the initiation of pro-oxidant and inflammatory responses [43,44].

In this respect, high glucose levels and AGEs formation is also present in diabetes, a risk factor for neurodegenerative disorders that induce damage to sensory neuron cytoskeleton proteins and decrease the ability of these neurons to extend neurites [45]. Considering this evidence, our data suggest that this model could also be further explored to study aspects of neurodegenerative diseases, such as Parkinson’s and Alzheimer’s diseases.

Sensory neuron activation can often cause pain hypersensitivity due to altered neurotransmitter release and disruption of neuron-to-neuron communication [41]. Substance P (SP) is a highly conserved member of the tachykinin peptide family that is involved in several neuronal signaling pathways, transmitting the nociceptive signals via primary afferent fibers to spinal and brainstem second-order neurons [46]. In our study, the results showing that ECM-GC induces substance P release clearly shows that these cells function as sensory-like neurons. Importantly, morphine, a known analgesic compound significantly decreases the ECM-GC-induced substance P release, confirming that this is a good model for searching new analgesic molecules.

Curiously, at timepoints 1 and 45 min, the release of substance P was not detected, suggesting that the glycation does not stimulate the release of neurotransmitters stocked at vesicles, but stimulates de novo substance P synthesis. Further experiments are necessary to test this hypothesis. Moreover, production of β-endorphin, an endogenous analgesic molecule released by both central and peripheral neurons [47], was unaffected by any of the treatments and incubation periods. Despite that, this parameter is useful in the screening of analgesic molecules.

Taken together, we developed a model combining sensory neuron-like cells derived from the human neuroblastoma SH-SY5Y cell line with ECM-GC that mimics a pro nociceptive microenvironment. The glycated collagen process is sufficient to increase c-Fos transcription factor expression, induce substance P release and inhibit neurite outgrowth, suggesting that ECM-GC induces neuronal activation. Furthermore, ECM-GC modulates Nav1.7 and NK1 gene expression in sensory neuron-like cells and may became a valuable tool for studying new analgesics candidates, as well as for the discovery of new therapeutic targets.

It is important to mention that the AGE effects on neuronal functions are temporary and delayed, which could be a limitation for screening models. However, considering that AGE induces cytoskeleton filaments reorganization and neurite retraction, alterations in the receptor expression and pronociceptive mediators may occur in later stages. Under this scenario, the late responses detected in our model may be relevant when evaluating chronic conditions.

For example, the severe diabetic neuropathy is poorly controlled in the patients, which represents a medical challenge [48]. Although the exact causes of its pathogenesis are unknown, increased AGEs may be involved on the neurodegeneration [49,50]. Besides high blood sugar that leads to nerve damage and impairs the nerves’ ability to transmit signals; high blood pressure, genetic factors, high triglyceride and cholesterol levels are also involved in the increased risk of neuropathy [49]. Unfortunately, the available treatments only control the glycemia and the pain symptoms. This model can be a valuable tool for screening candidates and/or molecular targets for the management of metabolic neuropathies.

Neurons derived from SH-SY5Y cells have several advantages—for example, it allows for large scale drug screening since they are easy to handle, are fast-growing, and easy to differentiate in neurons. However, the results found in neurons derived from SH-SY5Y cells should be validated and refined in human induced pluripotent stem cells (hiPSCs), for example.

Importantly, cell culture models may also serve as alternative methods for the replacement of animal use in research, in line with reducing waste, reusing, and recycling resources and products (3R principles) [51]. Finally, the development of novel cell culture models with physiological relevance may bridge the gap between current preclinical animal models and humans reduce the duration and costs, allowing the discovery of promising drug targets that can be tested in future clinical trials.

## 5. Conclusions

Collagen-derived advanced glycation endproducts acting in human sensory neuron-like cells mimic a painful microenvironment that positively responds to analgesics. Together, our results suggest that we established a functional model that can be useful as a platform for screening candidates for the management of painful conditions.

## 6. Patents

The in vitro glycation model of type I collagen, neuronal differentiation was subject to BR 102018008561–1 and BR102019008695-5, Title: “Process and Kit to identify molecular entities involved in the pain of osteoarthritis”.

## Figures and Tables

**Table 1:** Sequences of forward and reverse primers.

Genes	Primer Forward Sequence	Primer Forward Sequence
*HPRT1*	CCTGGCGTCGTGATTAGTGAT	AGACGTTCAGTCCTGTCCATAA
*PPIA*	GCCGAGGAAAACCGTGTACT	TGTCTGCAAACAGCTCAAAGGA
*SCN9A*	GTCTCCCTGGTTGATGGACG	TGATTGGTCGTGCCCTCTGG
*TACR1*	ATGACAGGTTCCGTCTGGGC	TACACACTGCCCTGGGTCTG

## Data Availability

All data from the study are given in the manuscript.

## Supplementary Materials

The following are available online at https://www.mdpi.com/2073-4409/11/2/247/s1, Figure S1: Cellular viability, Figure S2: Genes heat map analysis. Figure S3: Gene heatmap analysis.
