# Correction: Bufalo et al. Human Sensory Neuron-like Cells and Glycated Collagen Matrix as a Model for the Screening of Analgesic Compounds. *Cells* 2022, *11*, 247

**DOI:** 10.3390/cells13131089

**Published:** 2024-06-24

**Authors:** Michelle Cristiane Bufalo, Maíra Estanislau Soares de Almeida, José Ricardo Jensen, Carlos DeOcesano-Pereira, Flavio Lichtenstein, Gisele Picolo, Ana Marisa Chudzinski-Tavassi, Sandra Coccuzzo Sampaio, Yara Cury, Vanessa Olzon Zambelli

**Affiliations:** 1 Laboratory of Pain and Signaling, Butantan Institute, São Paulo 05503-900, Brazil; michelle.bufalo@butantan.gov.br (M.C.B.); gisele.picolo@butantan.gov.br (G.P.); 2 Center of Excellence in New Target Discovery, Butantan Institute, São Paulo 05503-900, Brazil; mesalmeida@gmail.com (M.E.S.d.A.); carlos.ocesano@butantan.gov.br (C.D.-P.); flavio.lichtenstein@butantan.gov.br (F.L.); ana.chudzinski@butantan.gov.br (A.M.C.-T.); 3 Laboratory of Pathophysiology, Butantan Institute, São Paulo 05503-900, Brazil; sandra.coccuzzo@butantan.gov.br; 4 Immunogenetics Laboratory, Butantan Institute, São Paulo 05503-900, Brazil; jose.jensen@butantan.gov.br; 5 Innovation and Development Laboratory, Innovation and Development Center, Butantan Institute, São Paulo 05503-900, Brazil; 6 Department of Pharmacology, Institute of Biomedical Sciences, University of São Paulo, São Paulo 05508-220, Brazil

## Error in Figure

In the original publication [1], there was a mistake in Figure 8, Similarity between Control and NC figures, as published. The corrected Figure 8 appears below. The authors state that the scientific conclusions are unaffected. This correction was approved by the Academic Editor. The original publication has also been updated.

## Figures and Tables

**Figure 8:**
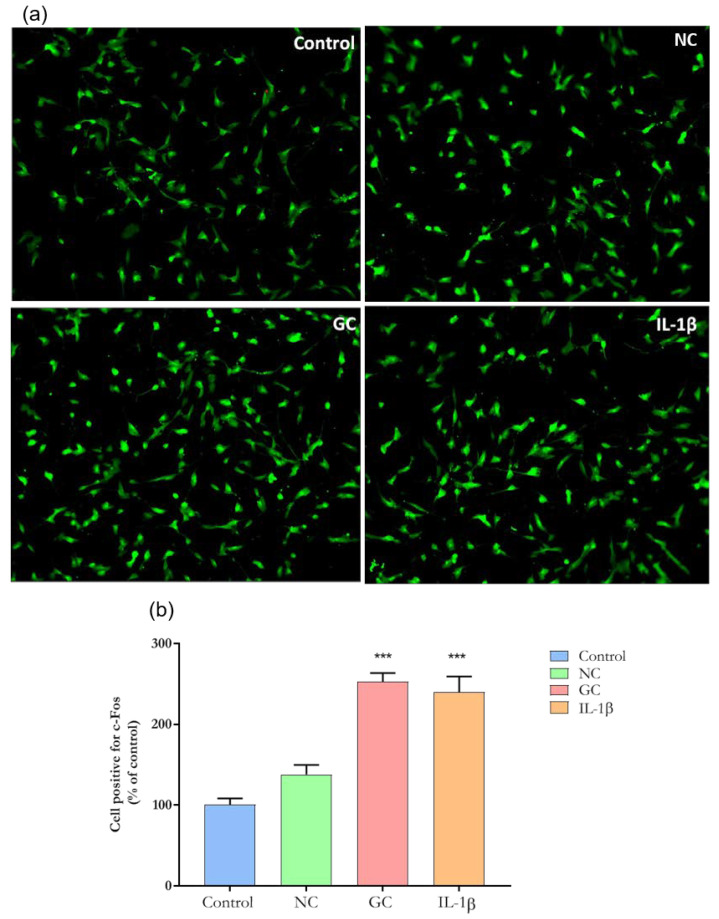
Representative images of immunofluorescence of c-Fos expression. (**a**) c-Fos expression in sensory neuron-like cells incubated with ECM-NC, ECM-GC, ECM-NC IL-1β or medium culture for 1 h. Magnification: 20×. (**b**) Quantification of c-Fos nuclear expression (% of control). Data were analyzed by one-way ANOVA with post hoc testing by Dunnett (*** *p* < 0.0001 different from Control). NC (normal collagen) and GC (glycated collagen). Three independent and similar assays performed in triplicate.
