# Supplementary material for: Human Sensory Neuron-like Cells and Glycated Collagen Matrix as a Model for the Screening of Analgesic Compounds
Source: Cells. 2022 Jan 12;11(2):247. doi: 10.3390/cells11020247 (PMC8773477; doi:10.3390/cells11020247)
Supplement: Supplementary file 1 [file cells-11-00247-s001.zip › cells-1534254-supplementary.pdf]

cells

MDPI

Supplementary Material

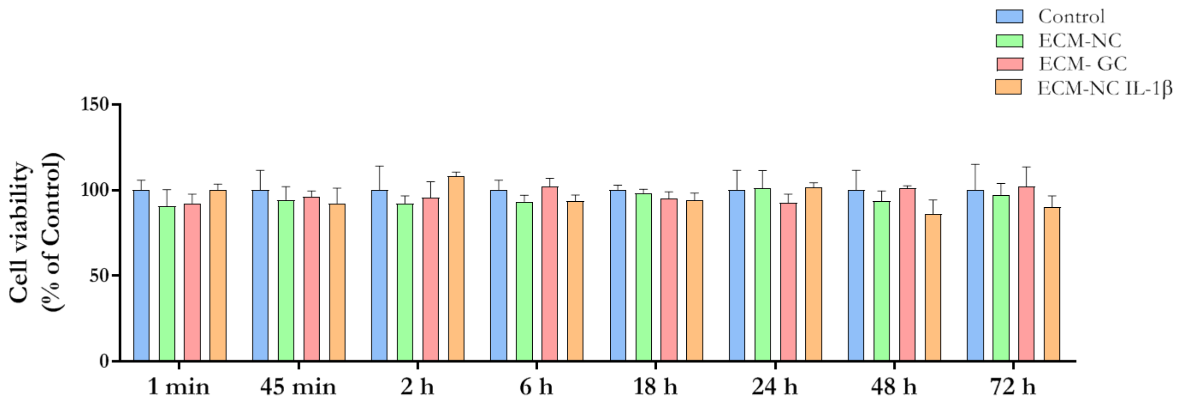

Supplementary Figure S1. Cellular viability. Cell viability (% of control) was performed at 1 min, 45 min, 2h, 6h, 18h, 24h, 48h, 72h. Two-way ANOVA and Dunnett post hoc test.

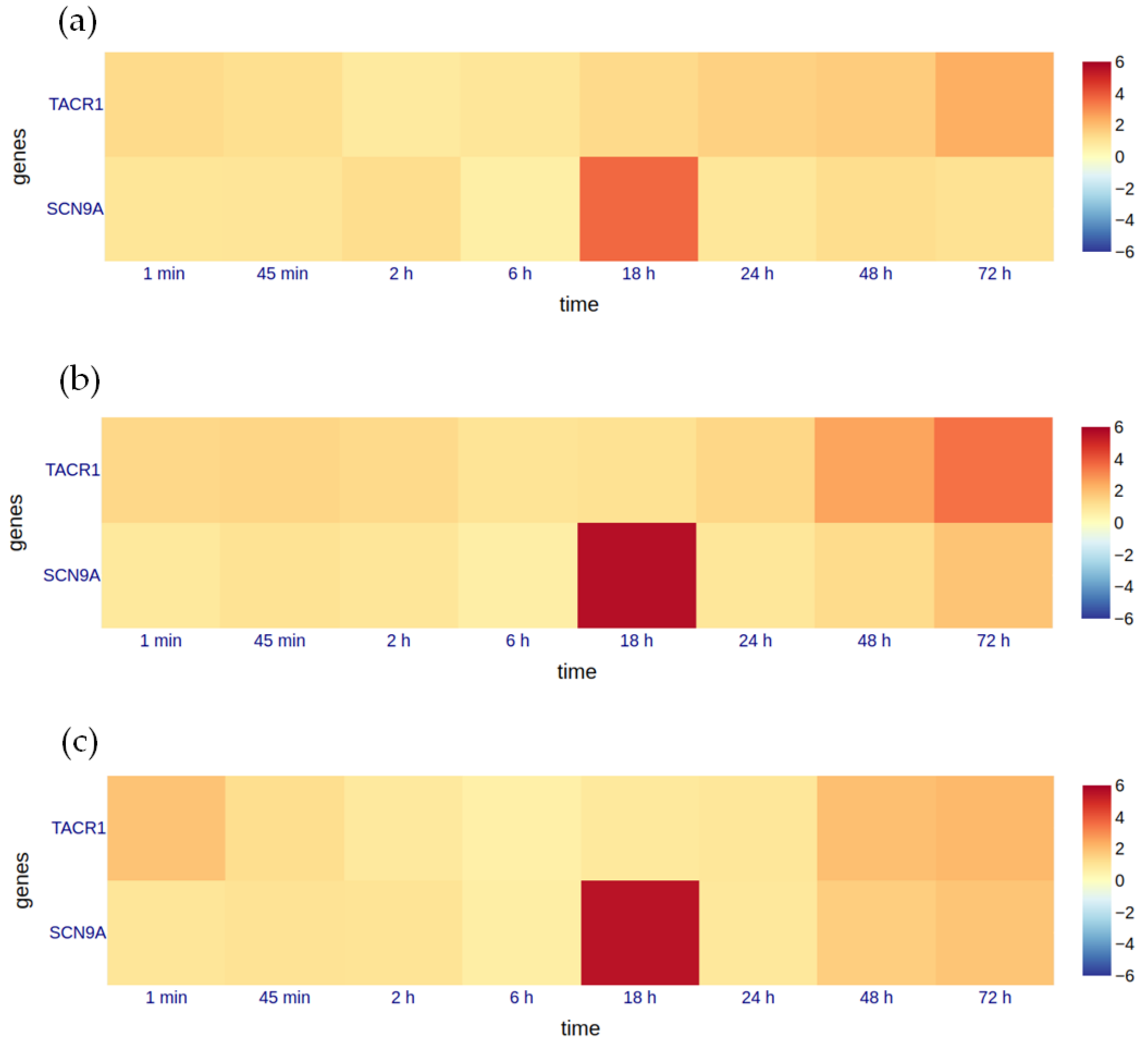

Supplementary Figure S2. Log fold change (LFC) time-series heatmap for *TACR1* and *SCN9A* related to 3 different conditions. (a) LFC (ECM-NC/control) the normal collagen, (b) LFC (ECM-GC/control) the glycated, and (c) LFC (ECM-NC IL-1β/control) the positive control. *TACR1* shows its maximum values at 72 h (LFC = 2.37 [2.18, 2.55], 3.52 [3.25, 3.79], and 2.15 [1.82, 2.47] for ECM-NC, ECM-GC, and ECM-NC IL-1β, respectively); in brackets the confidence interval for a

confidence level of 70 %. *SCN9A* shows its maximum values at 18 h (LFC = 3.71 [3.42, 3.99], 5.62 [5.41, 5.84], 5.53 [4.85, 6.20] for ECM-NC, ECM-GC, and ECM-NC IL-1β, respectively).

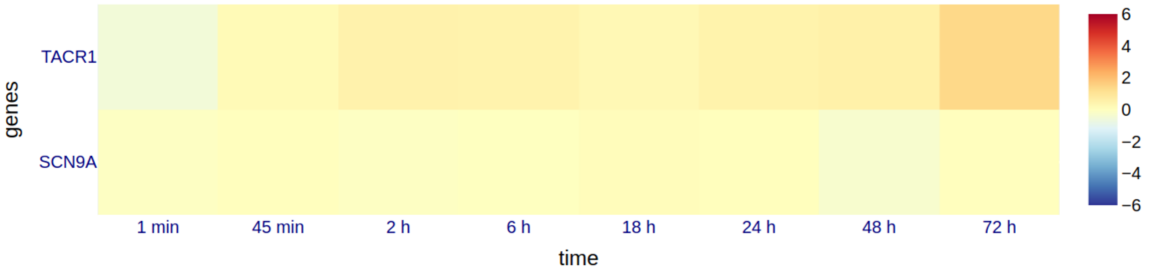

Supplementary Figure S3. LFC (ECM-GC) – LFC (ECM-NC IL-1β) time-series heatmap for *TACR1* and *SCN9A*, meaning how different are the glycated condition compared to the positive control condition (IL-β1). All values close to zero ( ]-1.2, 1.2[ ) represent similar LFC, (t-test *p*-value = 0.18 and n = 3).
